# The mediating role of social media disorder in the relationship between social media use and self-harm behavior in Indonesian adolescents

**DOI:** 10.3389/fpubh.2025.1663729

**Published:** 2025-10-08

**Authors:** Fransiska Kaligis, Cokorda Istri Agung Dewinta Adnyani, Kevin Girisamudra Wikanta, Muhammad Dzaky Darmawan, Muhammad Reza, Billy Pramatirta, Ruziana Masiran

**Affiliations:** ^1^Division of Child and Adolescent Psychiatry, Department of Psychiatry, Faculty of Medicine, Universitas Cipto Mangunkusumo Hospital, Jakarta, Indonesia; ^2^Medical Staff Group-Psychiatry, Universitas Indonesia Hospital, Depok, West Java, Indonesia; ^3^Faculty of Medicine, Universitas Indonesia, Jakarta, Indonesia; ^4^Faculty of Medicine and Health Sciences, Universiti Putra Malaysia, Serdang, Malaysia

**Keywords:** adolescent, mental health, self-harm, social media, disorder, mediating

## Abstract

**Introduction:**

Social media use in adolescence has been at an all-time high, along with the constant increase of self-harm behavior. Existing research on the relationship between social media usage and self-harm behavior in adolescent is inconsistent and scarce. We aimed to determine the relationship between adolescent self-harm behavior and social media usage, as well as to explore the mediating role of social media disorder in the relationship between social media use and self-harm behavior in Indonesian adolescents.

**Method:**

A cross-sectional study was conducted to investigate the relationship between self-harm behavior and social media use in 1096 adolescents aged 13–18 years old, who attend secondary and high schools in Jakarta.

**Results:**

Higher social media usage intensity was significantly correlated with increased instances of self-harm (*p* < 0.001). Similarly, a significant association was found between social media disorder and self-harm behavior (*p* < 0.001). Confirmatory Factor Analysis (CFA) yielded an acceptable and good fit of latent construct modeling based on several indices. Regression analysis indicated a strong link between social media usage time and social media disorder (*β* = 0.277, *p* = 0.035), and between social media disorder and self-harm behavior (*β* = 0.353, *p* < 0.001).

**Conclusion:**

Social media usage intensity and social media disorder was associated with self-harm behavior in adolescents, with social media disorder partially mediating the link between social media usage intensity and self-harm behavior. Our findings emphasize the importance of monitoring and managing social media use among adolescents to mitigate the risk of self-harm.

## 1 Introduction

The realm of social media has witnessed an unprecedented surge over the years, with active users doubling from 1.7 billion in 2013 to a staggering 4.76 billion in 2023. Social media has become an integral part of our daily routines, influencing our behaviors and interactions. Within this digital landscape, various user types emerge, distinguished by their engagement levels and behaviors, often intersecting with mental health considerations (1).

Self-harm, encompassing deliberate acts of harm without clear motives, presents a concerning issue, especially among adolescents. Shockingly, it ranks among the leading causes of adolescent mortality, closely intertwined with suicide as a significant risk factor. Insights from the Indonesia-National Adolescent Mental Health Survey (I-NAMHS) revealed a high prevalence of self-harm among adolescents, underscoring the urgency of addressing this issue (2–4).

The rise in social media usage coincides with an increase in self-harm occurrences, hinting at a potential correlation between the two phenomena. A survey in the United Kingdom found that around 93% of 12–17 year old adolescents had a social media profile (5). While previous studies have explored this relationship, a notable gap remains in understanding the specific factors mediating this association, particularly among adolescents (6).

Given the susceptibility of adolescents to self-harm, there is a pressing need for research focusing on this demographic. This study aims to investigate the correlation between problematic social media usage and self-harm behavior among Indonesian adolescents aged 13–18 years old, and to determine the direction of the correlation.

## 2 Methods

### 2.1 Study design

This study employed a cross-sectional research design to examine the association between self-harm behavior in adolescents aged 13–18 and problematic usage of social media. The quantitative questionnaires were distributed to junior and senior high schools of all grades (grade 7—12) in Jakarta for the purpose of this study.

### 2.2 Sample size

The sample size was taken based on the sample size formula for estimating the proportion of a population in community studies, to have a confidence level of 95 and 3% of the margin of error. Based on the formula, the minimum number of samples required is 1,068. However, 1,096 subjects who meet the inclusion criteria were participated in this study.

### 2.3 Recruitment and selection

Participants were recruited from public and private middle and high schools’ students aged 13–18 years old in Jakarta, Indonesia. All students from the selected schools were invited to join the study, but for those having physical or psychological conditions that rendered the participant unable to complete the questionnaire at the time of data collection were not included.

### 2.4 Data collection

The set of questionnaires given to subjects comprised of parental informed consent form, subject’s informed assent form, demographic information and questionnaires to assess self-harm behavior and social media usage. The demographic data categorizes parental income as below, equal to, or above the Provincial Minimum Wage (*Upah Minimum Provinsi* or UMP) of Jakarta province in Indonesia, set at IDR 4,901,798.00 (about USD 326), in accordance with Governor’s Decree Number 1153 of 2022. To ensure data privacy, participants were not required to provide their names or any other personal identifiers when completing the questionnaires. If subject wished to withdraw from the study, they could do so at any time by contacting the research team, either directly or through email.

The incidence and patterns of social media disorder, intensity of social media usage, and self-harm behavior among adolescents in Indonesia were examined using the Social Media Usage Intensity (SONTUS Questionnaire), Social Media Disorder (SMD) Scale, and Self-Harm Behavior Questionnaire (SHBQ). The Indonesian version of the Self-Harm Behavior Questionnaire (SHBQ) was employed to the subject as a self-report questionnaire containing five questions with follow-up questions divided into four sections to assess different behaviors: self-harm, suicide attempts, suicide threats, and suicidal ideation. This instrument was translated and validated into Indonesian language by Biromo (7) and has demonstrated good validity and reliability (Cronbach’s alpha = 0.94). Social media usage in the study was measured in terms of intensity and problematic use, with intensity measured using the Social Network Time Use Scale (SONTUS) translated into Indonesian language by Maria (8) with good validity and reliability (Cronbach’s alpha = 0.921), consisting of 29 questions measuring the time spent on social media. Social media use is categorized into low, average, high, and extremely high intensity. Problematic use of social media was identified with the Social Media Disorder (SMD) Scale, translated into Indonesian by Dewi and Lestari (9) with good validity and reliability (Cronbach’s Alpha = 0.734). The SMD scale assesses the history of social media problems in nine components: preoccupation, tolerance, withdrawal, persistence, displacement, problem, deception, escape, and conflict. Answering “yes” to more than 5 questions in the questionnaire indicates that the user is a problematic social media user.

### 2.5 Statistical analysis

Descriptive statistical analysis was performed using the Statistical Package for the Social Sciences (SPSS) on demographic data, social media usage intensity, problematic social media use, and self-harm behavior. The Confirmatory Factor Analysis (CFA) was conducted using the R software with the lavaan package. This method was chosen for its robust capabilities in psychometric validation and flexibility in modeling latent constructs. Model fit was assessed using several commonly reported goodness-of-fit indices, consistent with standards in psychometrics literature:

- Chi-square test: Assesses the overall fit of the model, with lower values and a non-significant *p*-value indicating a better fit. However, this test is sensitive to large sample sizes, which often result in significant values even for well-fitting models.
- Comparative Fit Index (CFI) and Tucker-Lewis Index (TLI): Both indices evaluate model fit relative to a null model, with values above 0.90 indicating an acceptable fit and values above 0.95 suggesting an excellent fit.
- Root Mean Square Error of Approximation (RMSEA): Reflects the model’s approximation to the data per degree of freedom, with values below 0.06 indicating a good fit.
- Standardized Root Mean Square Residual (SRMR): Represents the standardized difference between observed and predicted correlations, with values below 0.08 indicating a good fit.
- Adjusted Goodness of Fit Index (AGFI): Adjusts for model complexity, with higher values (preferably above 0.90) indicating better fit.

### 2.6 Ethical approval

This study received ethical approval from Faculty of Medicine Universitas Indonesia—Cipto Mangunkusumo Hospital Health Research Ethic Committee with approval number of KET-1655/UN2. F1/ETIK/PPM.00.02/2023 and ND-219/UN2. F1/ETIK/PPM.00.02/2024.

## 3 Results

### 3.1 Subjects characteristics

A total of 1,096 eligible adolescents completed the questionnaire. The median age of respondents was 15 years (range 13–18). Most of the respondents were female (58.2%) The sample socioeconomic status was diverse, with those below minimum wage making up the largest percentage of respondents (52.4%). Most of the respondents do not have any history of mental disorders (93.0%). The history of mental disorders reported by 77 respondents (7.0%) varied and included conditions such as anxiety, sleep disorders, depression, eating disorders, emotion disorders, etc. The subject characteristics can be seen in Table 1.

**Table 1:** Subject characteristics.

Variables	Total (*n* = 1,096)	Percentage (%)
Age (years old) (median ± min-max value)	15 (13–18)	
13	193	17.6%
14	252	23.0%
15	205	18.7%
16	237	21.6%
17	161	14.7%
18	48	4.4%
Gender		
Female	638	58.2%
Male	458	41.8%
Socioeconomic Status		
Above Minimum Wage	485	44.0%
Equal to Minimum Wage	40	3.6%
Below Minimum Wage	571	52.4%
History of Mental Disorders		
Yes (e.g., anxiety, sleep disorders, depression, eating disorder, emotion disorder, obsessive-compulsive disorder, and schizophrenia)	77	7.0%
No	1,019	93.0%
Social Media Usage Intensity		
Low	345	31.5%
Average	511	46.6%
High	213	19.4%
Extremely High	27	2.5%
Problematic Social Media User		
Yes	204	18.6%
No	892	81.4%
Self-Harm Behavior		
Ever	223	20.3%
Never	873	79.7%

### 3.2 Social media usage intensity in adolescents

Social media usage intensity from 1,096 subjects is obtained using the SONTUS questionnaire. The intensity is divided into 4 categories: low, average, high, and extremely high (Table 1). Of all subjects, almost half fall into the average category (46.6%). Only a small percentage of the subjects (2.5%) have extremely high social media usage intensity.

### 3.3 Problematic social media use in adolescents

Adolescents with problematic social media use are assessed using the Social Media Disorder Scale, where meeting more than 5 criteria classifies them as problematic users. Most of the respondents are not problematic social media users (81.4%). There are 204 adolescents (18.6%) who are classified as problematic social media users (Table 1).

### 3.4 Self-harm behavior in adolescents

A total of 223 (20.3%) adolescents reported having engaged in self-harm at least once in their life (Table 1). Follow-up questions regarding the characteristics of self-harm behavior were only answered by these 223 respondents (Table 2). From the demographic data, most of the respondents who engaged in self-harm are female, with a proportion of 83.0%. Most of the respondents, who have engaged in self-harm had socioeconomic status above the minimum wage (53.4%). The methods of self-harm that respondents used varied and included cutting themselves with sharp objects, hitting themselves, banging their heads against hard objects, pulling their own hair, hitting hard objects, pinching them-selves, gripping themselves, biting themselves, overdosing on medication, fasting for several days, and pouring hot water on themselves. Some of them also disclosed their specific method of self-harm. The most common method was cutting themselves on the wrist, thigh, and hand with sharp objects such as scissors, glass, and nails, which 135 (60.5%) respondents reported doing.

**Table 2:** Adolescent self-harm behavior characteristics.

Self-harm behavior	Total (*n* = 223)	Percentage
Gender		
Female	185	83.0%
Male	38	17.0%
Socioeconomic Status		
Above Minimum Wage	119	53.4%
Equal to Minimum Wage	13	5.8%
Below Minimum Wage	91	40.8%
History of Mental Disorders		
Yes	30	13.5%
No	193	86.5%
Self-harm behavior method*		
Cutting oneself (e.g., hand, wrist, thigh) with sharp objects (e.g., nails, scissors, broken glass, cutter)	134	
Hitting oneself	44	
Banging head against hard object	16	
Pulling own hair	16	
Hitting hard objects (e.g., wall, table, etc.)	13	
Pinching oneself	11	
Gripping oneself	10	
Biting oneself	9	
Overdosing on medication	5	
Fasting for several days	5	
Pouring hot water on oneself	1	
Not specifying the method	22	
Frequency of Self-Harm		
Once	31	13.9%
More than once	188	84.3%
Not Disclosed	4	1.8%
Age of first self-harm behavior		
Under 12 years	46	20.6%
12–16 years	167	74.9%
Over 16 years	6	2.7%
Not Disclosed	4	1.8%
Disclosure to others about self-harm		
Yes	77	34.5%
No	146	65.5%
Visited a doctor for self-harm behavior		
Yes	9	4.0%
No	214	96.0%

^*^Means: subject can choose more than one response.

Most of the respondents (84.3%) who had ever engaged in self-harm behavior have engaged in self-harm more than once, ranging from two to many or uncountable times, according to the responses from open question about the self-harm frequency. About age of first engagement in self-harm behavior, 167 (74.9%) respondents said between the ages of 12–16 years old, 46 (20.6%) respondents did it before turning 12 years old, 6 (2.7%) respondents did it after 16 years old, and 4 (1.8%) respondents did not disclose when they first engaged in self-harm. Most of the respondents did not tell anyone about their self-harm behavior. Only 77 respondents (34.5%) disclosed their self-harm behavior to others, especially their close ones like parents, best friends, girlfriends/boyfriends, or teachers. Only 9 (4.0%) of the respondents visited a doctor for their self-harm behavior.

### 3.5 Relationship between social media usage, social media disorder and self-harm behavior in adolescents

The Confirmatory Factor Analysis (CFA) yielded an acceptable model fit based on the following indices. The chi-square test statistic was significant (χ^2^(131) = 556.466, *p* < 0.001), which, while indicative of a lack of perfect fit, is common in large sample sizes. Additional goodness-of-fit measures demonstrated that the model performed well in approximating the data: Comparative Fit Index (CFI) = 0.907 and Tucker-Lewis Index (TLI) = 0.900, both exceeding the threshold for acceptable fit. The Root Mean Square Error of Approximation (RMSEA) was 0.054 (90% CI: 0.050–0.059), with *p*-values indicating that the RMSEA was below 0.08. The Standardized Root Mean Square Residual (SRMR) was 0.048, further supporting the model’s adequacy. The Adjusted Goodness of Fit Index (AGFI) of 0.925 suggested that the covariance structure of the data was well captured by the model. In terms of factor loadings, the standardized estimates showed strong associations between observed indicators and their latent constructs. Detailed regression coefficients and factor loadings are presented in Table 3, offering a comprehensive overview of the structural relationships.

**Table 3:** Detailed regression coefficients and factor loadings.

Latent variable	Indicator	Estimate	Std. all
SMU	Relaxation and free periods	17.252	0.961
	Academic-related periods	7.678	0.723
	Public-places-related use	7.182	0.720
	Stress related periods	8.280	0.585
	Motives for use	7.353	0.743
SMD	SMD1	0.207	0.443
	SMD2	0.228	0.505
	SMD3	0.233	0.501
	SMD4	0.187	0.399
	SMD5	0.176	0.379
	SMD6	0.155	0.336
	SMD7	0.172	0.414
	SMD8	0.163	0.370
	SMD9	0.164	0.454
SHBQ	Self-harm Behavior	2.668	0.626
	Suicide Attempts	1.248	0.472
	Suicide Threat	0.817	0.407
	Suicide Ideation	1.901	0.617

**Figure 1:**
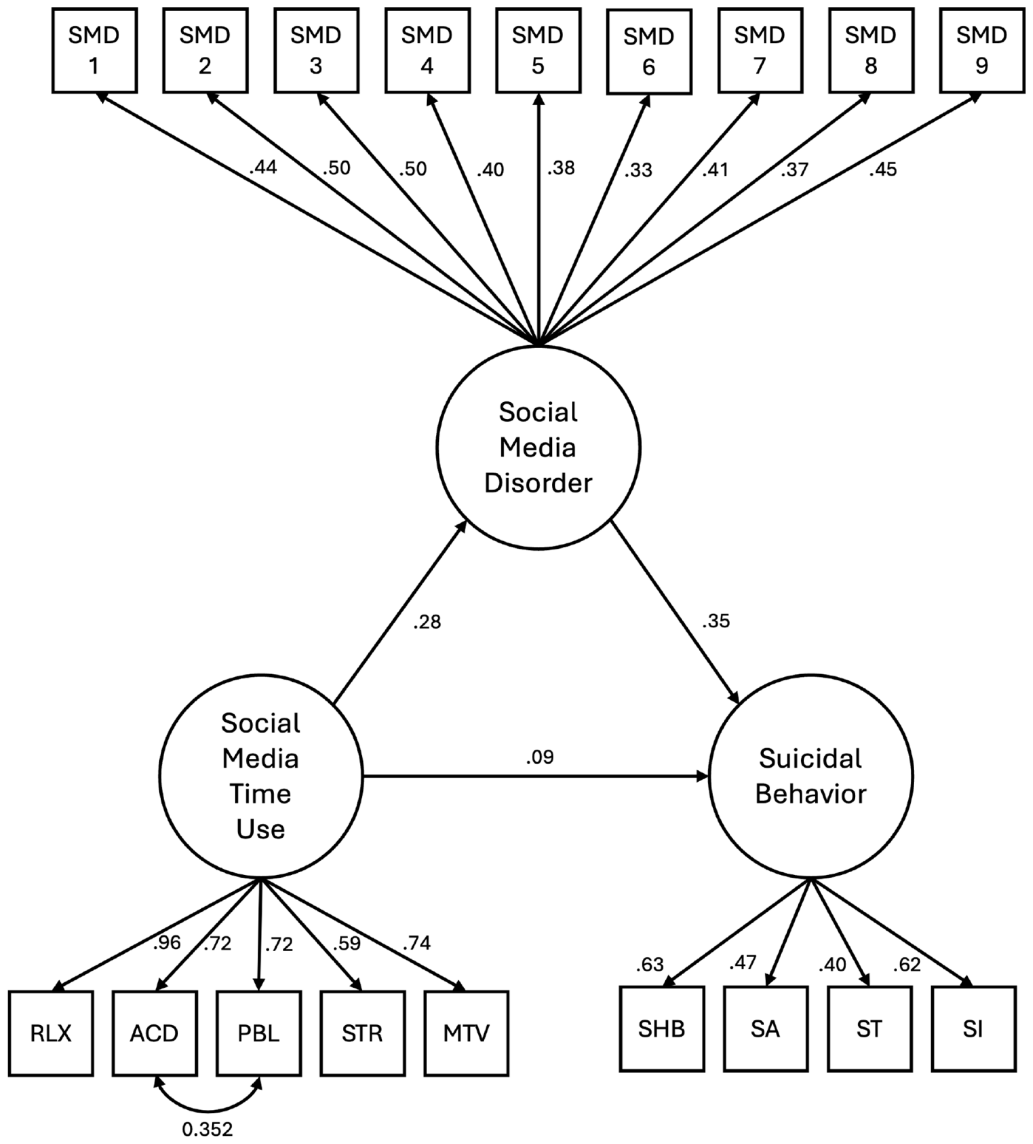
Relationship of suicidal behavior with social media use and social media disorder; NB: χ^2^(131) = 556.466, *p* < 0.001; CFI = 0.907; TLI = 0.900; RMSEA = 0.054; SRMR = 0.048; and AGFI = 0.925.

The regression results revealed significant relationships among the key constructs. Social media usage time was positively associated with self-harm behavior (*β* = 0.085, *p* = 0.035) and social media disorder (*β* = 0.277, *p* < 0.001). Furthermore, presence of social media disorder was significantly associated with presence of self-harm behavior (*β* = 0.353, *p* < 0.001). These findings suggest that social media disorder partially mediates the relationship between social media usage time and self-harm behavior. This highlights the complex interplay between the intensity and problematic aspects of social media use in influencing self-harming tendencies. Visual representation of the CFA model is provided in Figure 1, illustrating the relationships between latent variables (circles) and their observed indicators (rectangles), while arrows represent standardized factor loadings and correlations.

## 4 Discussion

The goal of our study is to determine the relationship between adolescents’ self-harm behavior and social media, indicated by social media usage intensity and social media disorder. We found that the social media usage intensity in Indonesian adolescents was mostly in the average-intensity group. This finding indicates that the frequency of social media use by adolescents does not exceed the average frequency of social media use by the general population (10). On the other hand, adolescents with high and extremely high intensity are often associated with mental health problems. High-intensity use of social media causes adolescents to reduce direct in-person contact and increases social isolation, stress, depression, and lack of sleep. The high-intensity social media usage also raises concerns about the risks that social media poses to adolescents, such as exposure to inappropriate content for their age, rude behavior from peers through negative comments, invasion of privacy, and bad influence from third parties (11).

Around 18.6% of respondents are classified as problematic social media users. Compared to the study by Boer, et al. (12) in 29 countries, the prevalence of problematic social media users in this study is relatively high (18.6%), exceeding the average prevalence in that study, 7.38%. Until now, no studies have examined problematic social media users among adolescents in Indonesia, so no comparable data is available. Adolescents become problematic social media users due to several factors, such as impulsivity, low self-esteem, emotional issues, and limited knowledge of potential social media problems. High levels of impulsivity cause attentional fluctuations, where individuals use social media to regulate emotions when things are out of their control. During adolescence, the ability to control impulses is still developing, thus increasing the risk of becoming a problematic social media user (13–15). Adolescence is also a critical developmental period in which often leads individual to an emotional crisis, causing insecurity and negative thoughts that leads to low self-esteem (13, 15). Adolescents with emotional issues, particularly social anxiety, tend to use social media to alleviate negative emotions by seeking attention, support, and acceptance (14). Finally, the limited knowledge of parents and people around adolescents about social media use can also influence the formation of problematic social media users (15).

Regarding self-harm, we found that the prevalence of adolescents who have engaged in self-harm behavior is 20.3%. In Asian countries, the prevalence of adolescents engaging in self-harm is around 19.5%, with a range of 17.1–22.2% (16). Xiao et al. (17) also found the prevalence of non-suicidal self-injury in non-clinical adolescent to be 22.0% (17.9–26.6%) in 2010–2021. We also found that most adolescents who engaged in self-harm behavior are female (83.0%). Another study also pointed out that females have a higher risk of self-harm due to associations with depression, eating disorders, and romantic relationship problems. Social factors also increase the risk of self-harm among females, as they tend to internalize negative emotions and self-harm can become a way to release or divert these negative emotions (2).

Self-harm is not a mental disorder but a failure to cope with stress (2). Coping mechanisms refer to the thoughts and behaviors used to manage both internal and external demands of stressful situations (18). In this study, 20.3% of adolescents have engaged in self-harm, indicating a failure in coping mechanisms. The environment can influence coping failure. An environment with maladaptive coping mechanisms, such as suppressing emotions, escaping from stress, and avoiding stressful situations, can lead adolescents to mimic these mechanisms. One such behavior is self-harm. High prevalence and exposure to self-harm among adolescents can encourage them to mimic and normalize this behavior (15).

Unfortunately, the majority of respondents (65.5%) never told anyone about their self-harm behavior. Research conducted by Klineberg et al. (19) found that adolescents who engage in self-harm are often reluctant to disclose their actions and have difficulty explaining their experiences. Many of the adolescents interviewed believed that self-harm is not something that needs to be shared and were concerned about the stigma from those around them. The fear of disclosing self-harm behavior also arises from the negative experiences of others when they have told someone about it. Fu et al. (20) found several negative perceptions from parents toward the behavior, including indifference, shame, and trivialization. Additionally, some parents’ initial responses to the behavior were negative, including blaming and indifference.

Furthermore, the number of adolescents who seek professional mental health support in this study was even lower (4%). This finding was consistent with the I-NAMHS study on Indonesian adolescents, which found that only 2.6% of adolescents with mental health problems would seek professional healthcare providers. The reasons adolescents do not seek professional help include preferring to solve the problem on their own, not knowing where to seek help, believing the problem will improve on its own, and the cost being too high (3). Adolescents also trust family and friends more than professional healthcare providers (21).

Our study showed a positive association between social media usage intensity and self-harm behavior in adolescents. There are several potential reasons for this relationship. First, social media serves as a place to seek social support from others suffering from similar mental health issues, where the content and information received can be negative, which potentially increases the desire or frequency of adolescents engaging in self-harm. Second, social media platforms contain content that increases exposure to and involvement in self-harming behavior, leading adolescents to adopt, be inspired or be encouraged to engage in self-harm, thus normalizing the behavior. Third, increased duration spent on social media can cause psychological stress, depression, and suicidal ideation in adolescents, which raises the likelihood of them engaging in self-harm (21). Another study on adolescents by O’Reilly et al. (10) reported that social media can cause depression and, in severe cases, lead to suicidal desires. Adolescents also reported that social media could increase the risk of engaging in self-harm due to the urge of imitating, cyberbullying, depression, and antisocial behavior.

We found a significant relationship between respondents’ problematic social media users and self-harm behaviors. This relationship indicates that problematic social media users have a higher risk of engaging in self-harm compared to respondents who are not problematic social media users. Several studies have established a significant relationship between problematic social media use and an increased risk of self-harm among adolescents.

A systematic review by Nesi et al. (22) highlights that adolescents who exhibit problematic social media use are more likely to experience suicidal ideation, which is a critical risk factor for self-harm behavior. In a study conducted in China, problematic social media users were found to be twice as likely to engage in self-harm compared to non-problematic social media users. This relationship was associated with adolescents’ failure to process impulses they received, leading them to release these impulses through self-harm (23). Similarly, a study by Kaess et al. (24) reported that the risk of self-harming behavior was three times higher among problematic social media users. The study suggested that excessive internet use might contribute to the development of maladaptive coping strategies, including self-harm, although it emphasized the need for further research to establish a definitive causal relationship. Moreover, Boer et al. (12) found a correlation between problematic social media use and lower levels of mental health, including higher rates of depression. This study posits that the resultant feeling of a lack of meaning in life could increase the likelihood of self-harming behavior among social media users.

However, the interplay between social media usage and social media disorder in affecting adolescent self-harm behavior is complex. Although we found both social media usage (SMU) intensity and social media disorder increase the likelihood of self-harm behavior in adolescents, the results of our study suggest social media disorder partially mediates the relationship between social media usage time and self-harm behavior. Previous studies showed conflicting results between the association of social media usage intensity and adolescents’ well-being. Although SMU intensity is closely associated with problematic SMU, it is not the same concept (25).

Problematic SMU is consistently related to lower well-being in adolescents (e.g., symptoms of depression, anxiety, body image dissatisfaction, irritability, nervousness, lower life-satisfaction and psychosocial well-being, and lower self-esteem) (25). Aside from self-harm behavior, several studies also highlighted the link between problematic SMU with suicidal ideation in adolescents and young adults (26, 27). In the present day, self-harm contents in social media are ubiquitous. A study showed that young people who get exposed to self-harm on Instagram, both intentionally or by accident, are at higher risk of self-harm and other suicide related outcomes (28). The effect of regularly viewing self-harm pictures on the social media can invoke physical reaction and inspire young people for their own self-harm behaviors. Frequently viewing these contents can normalize self-harm behavior in the eyes of the young people (29). Therefore, problematic SMU must be considered when investigating the relationship between SMU intensity and adolescents’ self-harm behavior (25). Our study added a critical insight that problematic SMU or social media disorder mediated the relationship between high SMU intensity and self-harm behavior in adolescent, indicating that high SMU intensity by itself does not directly cause increased risk of self-harm behavior in adolescent.

Another hypothesis that tried to explain the relationship between SMU intensity and adolescents’ mental health is the digital Goldilocks hypothesis. The hypothesis states that adolescents’ well-being up until a certain point, after which, any increase in screen time will be associated with decreased well-being. The assumption is that lack of SMU might impede young people from forming and sustaining social relationships and participating in social activities essential for their development. On the other side, excessive SMU might replace vital daily activities like attending in-person social gatherings, exercising, or completing schoolwork, negatively impacting adolescents’ well-being. Przybylski and Weinstein (30) found that the link between digital screen time (such as using computers, smartphones, social media, or gaming) and mental well-being is not linear, but curvilinear. They suggested that moderate digital activity is optimal. This hypothesis is further strengthened by the findings of Boniel-Nissim et al. (25) who found that non-active and problematic social media users had lower life satisfaction and lower levels of support from their peers compared to active social media users.

### 4.1 Strength and limitations

To date, only a few studies have explored the relationship between social media use and self-harm behavior in adolescents. In Jakarta, the prevalence of both self-harm and social media usage behavior is unknown. As the first study of its kind in the region, our research provides valuable baseline data for future investigations. Additionally, this is also the first study to use Confirmatory Factor Analysis (CFA) to ensure accurate measurement of social media disorder and self-harm behavior. This approach also minimizes measurement error and strengthens confidence in the structural relationships tested, particularly in understanding how social media usage and social media disorder contribute to self-harm behavior. By focusing on adolescents aged 13–18 years, particularly those in junior high and high school—an age group where self-harm behavior often emerges—this study offers crucial insights into the early impact of social media on mental health.

This research used a non-random sampling technique due to limited permission and time for data collection from the schools. In addition, this study only includes adolescents who attended school, so the findings may not be representative of those who did not attend school. Furthermore, this study was unable to explore the underlying reasons for self-harm or adolescents’ perceptions of this behavior. Social media use was assessed based on intensity and problematic usage, but factors such as the purpose of use, specific content consumed, and preferred social media platforms were not examined. Additionally, other factors which may be associated to self-harm were also not explored in this study, such as physical activity levels, social support availabilities, and the use of AI to seek for help which has increased recently.

## 5 Conclusion

We found that there is a significant relationship between social media usage intensity, problematic social media users, and self-harm behavior among adolescents. Adolescents with higher levels of social media usage intensity and those experiencing social media disorder are more likely to engage in self-harm behaviors. We also found that there is a stronger relationship between self-harm behavior and problematic social media users than social media usage intensity, with social media disorder partially mediated the link between social media usage intensity and self-harm behavior.

We recommend to use this knowledge to educate people, especially adolescents and parents, about the impact of social media on mental health. For further research, studies can be carried out with a cohort research design to assess the cause-and-effect relationship. Qualitative research, such as semi-structured interview and thematic analysis, can also be carried out to gain a deeper understanding from adolescents about the self-harm behavior they engaged in. Additionally, further research can delve deeper into the use of social media such as the content, purpose, and kind of social media used by adolescents. As our study include participants from Indonesia, a South-East Asia nation, we recommend that similar research be carried out in other geographical regions to allow for cross-cultural comparisons and broader generalizability of the findings.

## Data Availability

The data analyzed in this study is subject to the following licenses/restrictions: the dataset will be available upon request. Requests to access these datasets should be directed to Fransiska Kaligis, fransiska.kaligis@ui.ac.id.
